# Competing-risks analysis for evaluating the prognosis of patients with microinvasive cutaneous squamous cell carcinoma based on the SEER database

**DOI:** 10.1186/s12874-023-02109-x

**Published:** 2023-12-07

**Authors:** Suzheng Zheng, Shuping Xie, Hai Yu, Xi Duan, Yong He, Chichien Ho, Yang Wan, Tie Hang, Wenhui Chen, Jun Lyu, Liehua Deng

**Affiliations:** 1 Department of Dermatology, The First Affiliated Hospital of Jinan University and Jinan University Institute of Dermatology, Guangzhou, China; 2 School of Basic Medicine and Public Health, Jinan University, Guangzhou, China; 3 Department of Dermatology, Affiliated Hospital of North Sichuan Medical College, Nanchong, China; 4 Guangzhou Jnumeso Bio-Technology Co., Ltd, Guangzhou, China; 5 Chinese Academy of Inspection and Quarantine Greater Bay Area, Zhongshan, China; 6 Shanghai Aige Medical Beauty Clinic Co., Ltd. (Agge), Shanghai, China; 7 Department of Clinical Research, The First Affiliated Hospital of Jinan University, Guangzhou, China; 8 Guangdong Provincial Key Laboratory of Traditional Chinese Medicine Informatization, Guangzhou, China; 9 Department of Dermatology, The Fifth Affiliated Hospital of Jinan University, Heyuan, China

**Keywords:** Squamous cell carcinoma, SEER program, Prognosis, Microinvasive, Competing-risks model, Fine-Gray model

## Abstract

**Background:**

Utilizing the traditional Cox regression model to identify the factors affecting the risk of mortality due to microinvasive cutaneous squamous cell carcinoma (micSCC) may produce skewed results. Since cause-specific mortality can guide clinical decision-making, this study employed the Fine-Gray model based on the Surveillance, Epidemiology, and End Results (SEER) database to identify significant predictive variables for the risk of micSCC-related mortality.

**Methods:**

This study used the information of patients with micSCC who were listed in the SEER database during 2000–2015. Cox regression and Fine-Gray models were utilized for the multivariable analysis, and Gray’s test and the cumulative incidence function were used for the univariable analyses.

**Results:**

There were 100 patients who died from other reasons and 38 who died from micSCC among the 1259 qualified patients with micSCC. Most were female, white, married, had localized metastasis, etc. According to the univariable Gray’s test (*P* < 0.05), the cumulative incidence rate for events of interest was strongly associated with age, sex, marital status, American Joint Committee on Cancer staging, radiation status, summary stage, chemotherapy status, surgery status, and tumor size. Multivariable Cox regression analysis and multivariable competing-risks analysis indicated that age, tumor size, and income were independent risk variables for the prognosis of patients with micSCC. In both age and tumor size variables, the competing-risks model showed a slight decrease in the hazard ratio and a slight narrowing of the 95% confidence interval compared with the Cox regression model. However, this pattern is not evident in the income variable.

**Conclusions:**

This study established a Fine-Gray model for identifying the independent risk factors that influence the risk of mortality among patients with micSCC. This study uncovers that, in the context of competing risks, age, tumor size, and income serve as independent risk factors influencing the risk of mortality due to micSCC among patients. Our findings have the potential to provide more accurate risk assessments for patient outcomes and contribute to the development of individualized treatment plans.

**Supplementary Information:**

The online version contains supplementary material available at 10.1186/s12874-023-02109-x.

## Introduction

Cutaneous squamous cell carcinoma (cSCC) is the second most common type of nonmelanoma skin cancer behind basal cell carcinoma [1]. It accounts for 20% of all skin cancers, with 1 million cases diagnosed each year in the United States and an estimated 9000 deaths, trailing only basal cell carcinoma [2]. The currently known main pathogenic factor for cSCC is ultraviolet radiation exposure, but it is also related to chronic immunosuppressed state, exposure to ionizing radiation, chronic skin conditions, inherited genetic conditions, human papillomavirus infection, chronic arsenic exposure, and other susceptibility factors [3–7]. cSCC is often localized and can therefore easily be treated using straightforward surgery or other local techniques. Invasive squamous cell carcinoma has a poorer prognosis than in situ squamous cell carcinoma because the tumor cells are more metastatic and invasive, and they can reach deeper tissues by breaking through the dermal papillary layer [8]. Although cSCC has a favorable prognosis overall, several aggressive variants can markedly increase the mortality risk. The risk factors impacting the prognosis of patients with microinvasive cSCC (micSCC) need to be fully identified since this is an aggressive subtype of cSCC.

Most previous studies on the prognostic factors of cSCC used conventional analysis techniques when examining several possible factors, such as the log-rank test, Cox regression model, and Kaplan–Meier estimation of patient survival probability [9–13]. A literature search did not reveal any previous analyses of the prognostic risk factors of invasive subtypes of cSCC. Patient deaths due to any cause other than cancer were often ignored by traditional analysis methods. There is a competitive relationship between death factors, and so previous analyses of potential factors have not been sufficiently accurate. When examining the variables that influence a cancer patient's prognosis, we should consider deaths caused by noncancer factors as competing-risks events and concentrate on deaths caused by specific cancer.

The Fine-Gray model is based on the principle of competing risks and is used to study the influence of risk factors on each type of event when there are many different possible events (such as death or failure). The Fine-Gray model utilizes subdistribution hazard ratios to quantify the relative effects of covariates on the subdistribution hazard function [14]. As such, covariates in the model can be interpreted as influencing the cumulative incidence function, or the probability of an event occurring over time. This is a departure from the traditional Cox regression model, which primarily focuses on the survival function, or the probability of survival until a given time point. In contrast, the Fine-Gray model emphasizes the cumulative incidence function, or the probability of a specific event occurring by a given time point. This distinction allows the Fine-Gray model to more accurately describe the occurrence of events in scenarios where competing risks are present. The Fine-Gray model is an extension of the traditional Cox proportional hazards model, which models different types of competing events by introducing a subdistribution model [15]. The Fine-Gray model provides subdistribution hazard ratios that consider both the cumulative incidence of the specific event and the presence of competing events, making it a superior choice when competing risks are involved. This accounts for complexities and prevents bias, making it a more accurate estimation method compared to the Cox regression model, which only provides hazard ratios without considering competing events. Compared with other traditional survival analysis techniques, the Fine-Gray model has the advantage of being able to more accurately assess the risk of the main outcome and solve the problem of different baseline risks under the premise of considering competing risks [16]. In the presence of competing risks, Cox regression model estimates can be biased, underestimating or overestimating the risk of a particular event [17]. Therefore, the Fine-Gray model can be used to identify the factors that influence the prognosis of patients with micSCC in order to exclude the impact of other causes of death on the accuracy of estimations. And comparing the results with the findings of classical analysis methods can more accurately reflect the real impact of variables in order to identify the relevant risk factors.

The Surveillance, Epidemiology, and End Results (SEER) database, which was founded in 1973 by the National Cancer Institute, serves as one of the most comprehensive major tumor databases in the US [18, 19]. It has compiled data on the mortality, prevalence, incidence, and other evidence-based medical data of patients with tumors over the course of several decades in many states and counties in the US. In this study, data were retrieved from individuals in the SEER database who had been diagnosed with micSCC, and the competing-risks analysis was carried out on the basis of considering the causes of death caused by micSCC and other causes, and the findings were compared with those obtained using the Cox regression model to determine the risk variables that influence the prognosis of patients with micSCC. This is the first study to examine factors associated with prognosis in patients with micSCC, with the aim of improving patient prognosis and providing decision making when individualizing treatment.

## Resources and methods

### Database

SEER*stat software was used to extract information of patients with a micSCC diagnosis from the SEER database (version 8.4.0.1). The SEER program was launched in 1974. This database, which comprises information from numerous places and encompasses demographic, staging, therapy, survival time, and other information, provides data for nearly 50% of the US population. It is therefore a reliable data source for assessing the incidence, prevalence, and survival rate of cancer in the US over time [20].

### Data collection and analysis

We extracted all data with ICD-O-3 “hist/behave” code 8076/3 (“squamous cell carcinoma, micro-invasive”) in the SEER database. The inclusion criteria were (1) year of diagnosis of 2000–2015 and (2) behavior code in ICD-0–3 of malignant. The exclusion criteria were as follows: (1) only autopsy results were available, (2) not the first primary sign of malignancy, (3) variables with incomplete information.

**Fig. 1:**
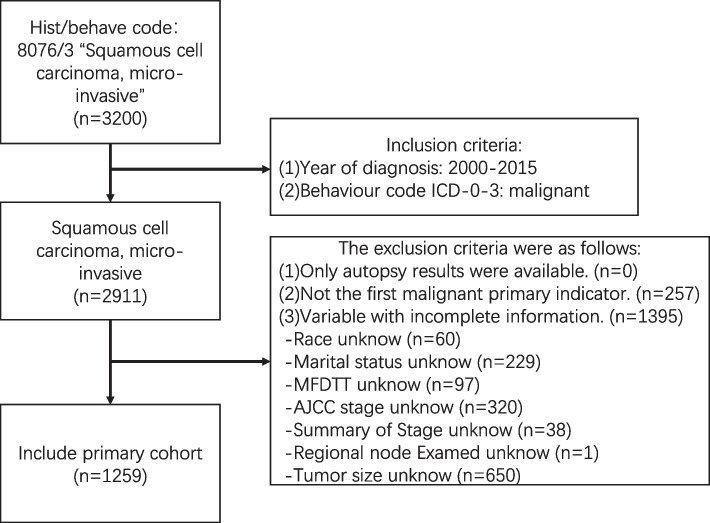
The procedure for including and excluding study participants

The choice of variables was guided by established melanoma research and clinical understanding, ensuring that selected factors were known or suspected to affect prognosis in microinvasive squamous cell carcinoma. This approach ensured that our analyzes focused on variables with proven or potential clinical significance [21, 22]. By thoroughly examining the interrelationships among covariates, including the utilization of variance inflation factors and correlation coefficient matrices to assess collinearity, our aim was to mitigate the potential collinearity risks that could impact the stability and interpretability of our model. The following data were gathered for each enrolled patient: age, sex, marital status, race, American Joint Committee on Cancer (AJCC) stage, summary stage (localized, regional, or distant), radiation status, chemotherapy status, surgery status, tumor size, regional lymph nodes removal, regional nodes examined, income, cause of death, survival months, vital status, and months from diagnosis to treatment (MFDTT). There were ultimately 1259 cases that met the above criteria, as depicted in Fig. 1.

### Statistical evaluation

Frequencies and percentages were used to portray categorical data, whereas the mean and standard deviation were used to describe continuous data. The follow-up outcomes for all patients were separated into the following three groups according to the vital status recode and cause-specific death classification in the SEER database in order to construct the Fine-Gray model: cancer deaths, censored, and non-cancer deaths. The cumulative incidence function (CIF) allows for the respective CIF of events of interest and the CIF of competing events. The CIF assumes that only one event occurs each time, and has the expected attribute that the sum of the CIFs among all categories is equal to the CIF of the composite event [23]. The expression is CIFk(t) = Pr(T ≤ t, D = k). The likelihood of each occurrence was determined using the CIF as a univariable analysis. Nelson-Aalen cumulative risk curves were then produced to determine the incidence function for micSCC-specific mortality, and the outcomes were compared between the two groups using Gray’s test [24, 25]. Based on the results of the univariable analysis and clinical relevance of variables, the Fine-Gray and Cox regression models were used for the multivariable analysis, and their results were compared to ascertain the realistic factors that affected the prognosis of patients with micSCC.

The R package and IBM SPSS software (version 27.0, SPSS, Chicago, IL, USA) were used for all data analyses in this study. The inspection level was α = 0.05, with *P* < 0.05 indicating significance, and all tests were two-sided.

## Results

### Patient characteristics

The basic information of the patients is listed in Table 1. The 1259 suitable patients with micSCC were aged 44.57 ± 13.55 years (mean ± SD) and their MFDTT was 0.54 ± 1.15 months. Most were female (*n* = 1197, 95.08%), white (*n* = 1001, 79.51%), married (*n* = 642, 50.99%), in AJCC stage I (*n* = 1194, 94.84%), had localized metastasis (1203, 95.56%), did not receive radiotherapy or had an unknown radiotherapy status (*n* = 1190, 94.52%), did not receive chemotherapy or had an unknown chemotherapy status (*n* = 1215, 96.51%), had received surgery (*n* = 1206, 95.79%), had unresected regional lymph nodes (*n* = 1123, 89.20%), had a tumor size of < 20 mm (*n* = 1180, 93.73%), had not received a regional lymph node examination (*n* = 1057, 83.96%), and had an income of US$ 55,000–75,000 (*n* = 744, 59.09%). Among all included patients, 100 (7.94%) died from other causes, while micSCC was the cause of death in 38 individuals (3.02% of all enrolled patients). The individuals who died of micSCC were more likely to be middle-aged, male, black, separated/divorced/widowed, and have AJCC stage IV, distant metastasis, received radiotherapy and chemotherapy, no surgery, regional lymph node resection, tumor size > 40 mm, regional lymph node examination, income > $75,000, and late treatment. The median follow-up time among all patients was 156 months.

**Table 1:** Patients characteristics and demographics

Variables	Total (*n* = 1259)	Cause-specific death (%)	Death due to other causes (%)	Median	Min	Max	Q1	Q3
**Age**	44.57 ± 13.554	55.87 ± 17.781	59.6 ± 15.165	42	17	95	34	53
**Sex**								
Male	62	7 (11.290%)	13 (20.968%)					
Female	1197	31 (2.590%)	87 (7.268%)					
**Race**								
White	1001	31 (3.097%)	78 (7.792%)					
Black	119	4 (3.361%)	15 (12.605%)					
Other	139	3 (2.158%)	7 (5.036%)					
**Marital status**								
Married	642	12 (1.869%)	36 (5.607%)					
Single	383	11 (2.872%)	27 (7.050%)					
DSW	234	15 (6.410%)	37 (15.812%)					
**AJCC stage**								
I	1194	23 (1.926%)	87 (7.286%)					
II	30	2 (6.667%)	9 (30.000%)					
III	25	8 (32.000%)	2 (8.000%)					
IV	10	5 (50.000%)	2 (20.000%)					
**Summary of Stage**								
Local	1203	23 (1.912%)	94 (7.814%)					
Regional	48	12 (25.000%)	4 (8.333%)					
Distant	8	3 (37.500%)	2 (25.000%)					
**Radiation**								
Yes	69	16 (23.188%)	16 (23.188%)					
No/unknow	1190	22 (1.849%)	84 (7.059%)					
**Chemotherapy**								
Yes	44	14 (31.818%)	6 (13.636%)					
No/unknow	1215	24 (1.975%)	94 (7.737%)					
**Surgery**								
Yes	1206	26 (2.156%)	90 (7.463%)					
No/unknow	53	12 (22.642%)	10 (18.868%)					
**Regional lymph nodes removal**								
Yes	136	6 (4.412%)	5 (3.676%)					
No	1123	32 (2.850%)	95 (8.459%)					
**Tumor size**								
≤ 2 cm	1180	21 (1.780%)	90 (7.627%)					
2 < X ≤ 4	43	7 (16.279%)	8 (18.605%)					
> 4	36	10 (27.778%)	2 (5.556%)					
**Regional node Examed**								
Yes	202	7 (3.465%)	9 (4.455%)					
No	1057	31 (2.933%)	91 (8.609%)					
**Income**								
< $55,000	199	3 (1.508%)	20 (10.050%)					
$55,000 ~ $75,000	744	23 (3.091%)	60 (8.065%)					
> $75,000	316	12 (3.797%)	20 (6.329%)					
**MFDTT**	0.54 ± 1.152	0.71 ± 1.063	0.54 ± 0.968	0	0	11	0	1

*DSW* divorced, separated or widowed

*MFDTT* months from diagnosis to treatment

### Univariable analyses of the micSCC prognosis

**Fig. 2:**
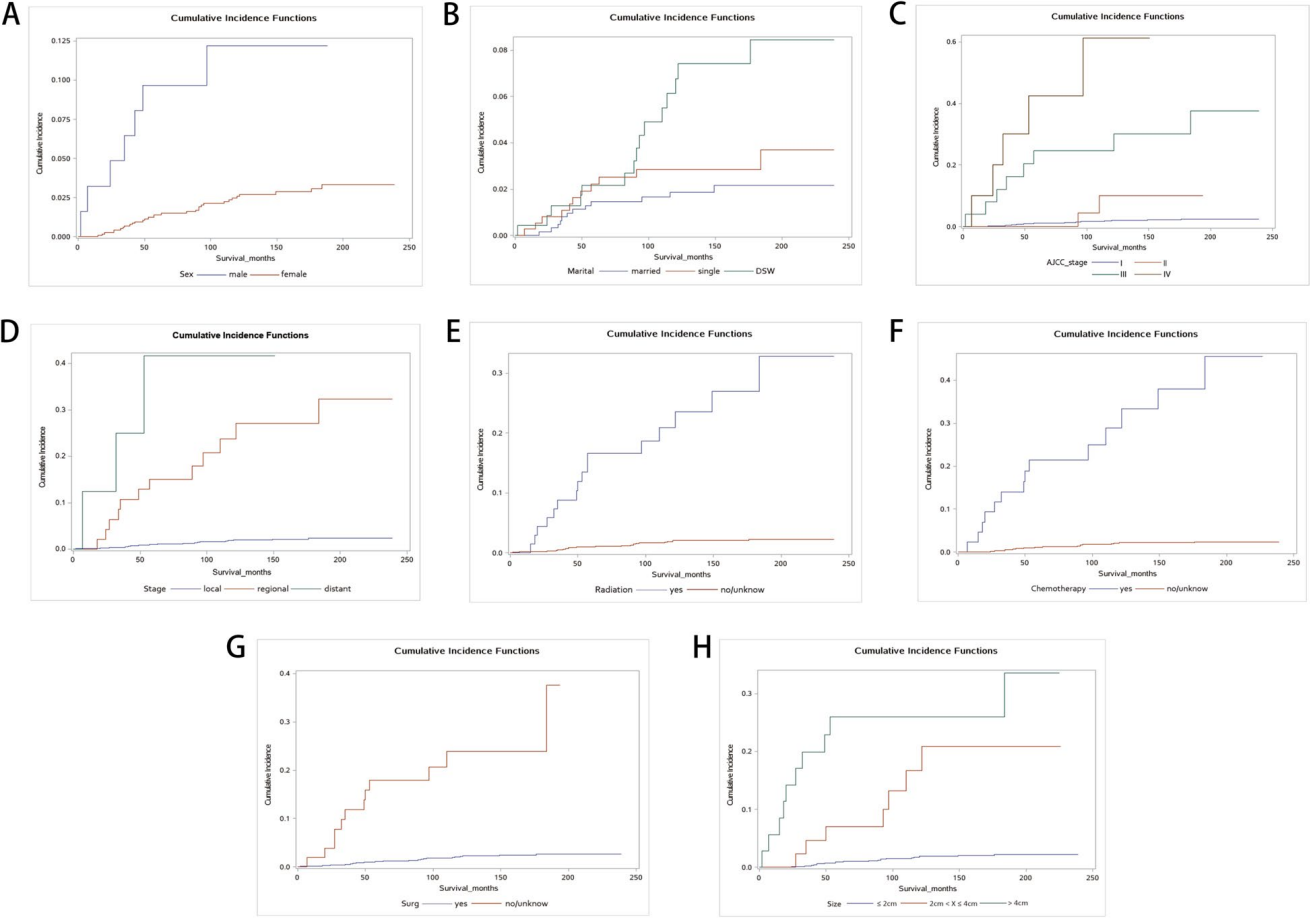
**A** Cumulative incidence curves of cause-specific death subgrouped by sex. **B** Cumulative incidence curves of cause-specific death subgrouped by marital status. **C** Cumulative incidence curves of cause-specific death subgrouped by AJCC. **D** Cumulative incidence curves of cause-specific death subgrouped by stage. **E** Cumulative incidence curves of cause-specific death subgrouped by radiation. **F** Cumulative incidence curves of cause-specific death subgrouped by chemotherapy. **G** Cumulative incidence curves of cause-specific death subgrouped by surgery. **H** Cumulative incidence curves of cause-specific death subgrouped by size

Univariable analyses using Gray’s test were performed on 14 potential prognostic factors, revealing that age, marital status, sex, AJCC stage, metastasis, radiation status, chemotherapy status, tumor size, and surgical status exhibited significant impacts on the prognosis of micSCC patients. The Nelson-Aalen cumulative risk curve of multicategorical variables is shown in Fig. 2. The CIFs of almost all variables showed upward trends at 1, 3, and 5 years. CIF values were higher for AJCC stage IV, distant metastasis, and large tumors. Table 2 presents a detailed list of the outcomes of the univariable analyses and CIF values.

**Table 2:** Univariable analysis of prognostic factors in patients with micSCC

			CIF		
Variable	Gray’s test	*p*-value	12-months	36-months	60-months
**Age**	376.791	< 0.0001			
**Sex**	18.543	< 0.0001			
Male			0.032	0.065	0.097
Female			0.000	0.007	0.014
**Race**	0.494	0.7813			
White			0.002	0.010	0.019
Black			0.000	0.000	0.017
Other			0.000	0.015	0.015
**Marital status**	11.918	0.0026			
Married			0.000	0.008	0.015
Single			0.003	0.011	0.022
DSW			0.004	0.013	0.022
**AJCC Stage**	166.006	< 0.0001			
I			0.000	0.004	0.010
II			0.000	0.000	0.000
III			0.040	0.162	0.246
IV			0.100	0.300	0.425
**Stage**	129.332	< 0.0001			
Local			0.001	0.004	0.010
Regional			0.000	0.107	0.151
Distant			0.125	0.250	0.417
**Radiation**	109.697	< 0.0001			
Yes			0.000	0.089	0.166
No/unknow			0.002	0.005	0.010
**Chemotherapy**	143.859	< 0.0001			
Yes			0.023	0.140	0.214
No/unknow			0.001	0.005	0.011
**Surgery**	83.507	< 0.0001			
Yes			0.001	0.005	0.011
No/unknow			0.019	0.118	0.180
**Regional lymph nodes removal**	1.240	0.2655			
Yes			0.000	0.015	0.031
No			0.002	0.009	0.017
**Tumor size**	111.472	< 0.0001			
≤ 2 cm			0.000	0.003	0.009
2 < X ≤ 4			0.000	0.047	0.070
> 4			0.056	0.199	0.260
**Regional node Examed**	0.095	0.7577			
Yes			0.000	0.010	0.021
No			0.002	0.010	0.018
**Income**	1.548	0.4612			
< $55,000			0.005	0.005	0.005
$55,000 ~ $75,000			0.000	0.011	0.023
> $75,000			0.003	0.010	0.016
**MFDTT**	10.656	0.3849			

*DSW* divorced, separated or widowed

*MFDTT* months from diagnosis to treatment

### Results from the multivariable analysis

The significant factors and clinically relevant variables in the univariable analyses were included in the multivariable Cox regression and the Fine-Gray model (*P* < 0.05). The analysis results from both models demonstrate that age, tumor size, and income achieved statistical significance. In both age and tumor size variables, the competing-risks model showed a slight decrease in the hazard ratio and a slight narrowing of the 95% confidence interval compared with the Cox regression model. However, this pattern is not evident in the income variable. The outcomes from the multivariable analysis of the Cox regression and Fine-Gray model are listed in Table 3.

**Table 3:** Two models of prognostic factors in patients with micSCC were multivariablely analyzed

	Cox model			Fine-gray model		
Prognostic factors	*P*-value	HR	95%CI	*P*-value	HR	95%CI
**Age**	< 0.0001	1.069	1.037–1.101	0.0005	1.059	1.026–1.094
**Sex**						
Male		reference			reference	
Female	0.6146	0.729	0.213–2.492	0.7458	0.775	0.166–3.620
**Race**						
White		reference			reference	
Black	0.9349	0.950	0.279–3.232	0.9312	1.058	0.297–3.770
Other	0.1216	0.308	0.070–1.367	0.2548	0.370	0.067–2.049
**Marital status**						
Married		reference			reference	
Single	0.0588	2.538	0.966–6.668	0.1503	2.149	0.758–6.093
DSW	0.1184	2.111	0.826–5.393	0.2340	1.894	0.662–5.418
**AJCC stage**						
I		reference			reference	
II	0.0503	0.127	0.016–1.003	0.1665	0.238	0.031–1.817
III	0.6348	0.607	0.077–4.772	0.7664	1.459	0.121–17.669
IV	0.2782	5.366	0.257–111.839	0.1146	15.720	0.513–481.776
**Summary of Stage**						
Local		reference			reference	
Regional	0.1179	3.883	0.709–21.263	0.5508	2.323	0.146–37.029
Distant	0.7520	0.579	0.020–17.174	0.6178	0.273	0.002–44.879
**Radiation**						
Yes		reference			reference	
No/unknow	0.4247	0.501	0.092–2.731	0.7637	0.667	0.048–9.338
**Chemotherapy**						
Yes		reference			reference	
No/unknow	0.3874	0.448	0.072–2.768	0.3931	0.379	0.041–3.514
**Surgery**						
Yes		reference			reference	
No/unknow	0.6010	0.678	0.158–2.907	0.5928	0.592	0.086–4.049
**Regional lymph nodes removal**						
Yes		reference			reference	
No	0.4175	0.396	0.042–3.722	0.4868	0.418	0.036–4.879
**Tumor size**						
≤ 2 cm		reference			reference	
2 < X ≤ 4	0.0288	4.871	1.178–20.145	0.0270	4.422	1.185–16.509
> 4	0.0003	15.717	3.485–70.883	0.0021	12.003	2.466–58.424
**Regional node Examed**						
Yes		reference			reference	
No	0.4824	2.101	0.265–16.680	0.5593	1.914	0.216–16.922
**Income**						
< $55,000		reference			reference	
$55,000 ~ $75,000	0.0036	16.573	2.499–109.930	0.0045	17.596	2.431–127.369
> $75,000	0.0071	13.224	2.020–86.584	0.0104	13.939	1.858–104.587
**MFDTT**	0.1606	0.743	0.491–1.125	0.2063	0.790	0.547–1.139

*DSW* divorced, separated or widowed

*MFDTT* months from diagnosis to treatment

## Discussion

This is the first study to explore factors associated with the prognosis of patients with micSCC. This study used information obtained from the SEER database to perform a competing-risks analysis in order to identify the risk factors that influence the prognosis of individuals with micSCC. The Fine-Gray model indicated that age, tumor size, and income were independent risk factors for specific death of individuals with micSCC.

Whether micSCC or other factors lead to the death of patients, they do not independently affect the outcome, because death due to one cause will inevitably lead to other factors not having an effect. This is contrary to the assumption that Cox regression analysis is based on. Direct analysis using Cox regression analysis may therefore produce incorrect analysis results and HR values. The Fine-Gray model can therefore more accurately identify the risk factors that influence the prognosis of patients when compared with the Cox regression model. The analysis results in this study demonstrated how markedly different the outcomes of the Fine-Gray and Cox regression models were. While both Cox regression and competing-risks analysis identified age, tumor size, and income as statistically significant factors, there were variations in the risk estimation results for these variables. Regarding the remaining eleven factors, both Cox regression and competing-risks analyses did not reveal significant effects on patient prognostic factors.

Both the Cox regression model and the Fine-Gray model take into account patient age as a factor that may affect prognosis. In a study on risk factors and prognosis of metastatic cSCC by Knuutila et al. [26], a binary logistic regression analysis indicated that age, metastatic characteristics, maximum lymph node metastasis, and AJCC-8 lymph node stage were found to be factors related to a poor prognosis. Similarly, age has been regarded to be a risk factor for the prognosis of patients in numerous studies, and many academics have acknowledged the significance of age in patient prognoses [27, 28]. In our analysis, we observed that the Cox regression analysis yielded relatively higher risk estimates, while the results of the competing-risks analysis displayed a tendency towards potentially more conservative risk assessments. (Cox regression model: HR = 1.069, CI = 1.037–1.101, *P* ≤ 0.05; Fine-Gray model: HR = 1.059, CI = 1.026–1.094, *P* ≤ 0.05). With the increase of age, cell senescence leads to the decline of its gene stability, and the chance of gene mutation increases, so the possibility of suffering from micSCC increases. At the same time, the body's response and tolerance to treatment are also declining during the aging process, which affects the survival of elderly patients. In recent years, the rapid development of gene-specific immune and targeted therapy is expected to further improve the survival rate of elderly patients, which is also a current and future research hotspot [29].

Tumor size is one of the basic indicators for assessing tumor malignancy or cancer staging, and it is also an important reference factor for clinicians to choose appropriate treatment options [30]. Its significance is self-evident. Many studies found that tumor size can affect tumor recurrence and metastasis, thus affecting the prognosis of patients [31–34]. The systematic reviews and meta-analyses by Zeng et al. [35], Thompson et al. [36], and Lubov et al. [27] all found that tumor size could affect the prognosis of patients with micSCC, which was consistent with the results of our analysis. Our competing-risks analysis results indicated that the survival risk for tumors with a size of > 2 cm and ≤ 4 cm was 3 times higher than that for tumors with a size of ≤ 2 cm (HR = 4.422, CI = 1.185–16.509, *P* ≤ 0.05), and that the survival risk for tumors with a size of > 4 cm was 11 times higher than that for tumors with a size of ≤ 2 cm (HR = 12.003, CI = 2.466–58.424, *P* ≤ 0.05), which indicates that patients with larger tumors have a worse prognosis. In comparison to the Fine-Gray model, the Cox regression model presents a marginally elevated HR value alongside a broader 95% confidence interval. These distinctions likely arise from the Cox regression model's exclusive emphasis on event timing, while the Fine-Gray model takes into account competing risks, including non-cancer-related mortality. Melanoma is more prevalent among the elderly population, and elderly cancer patients often face a higher likelihood of experiencing competing events, including conditions like cardiovascular disease and diabetes. Considering the potential impact of these competing events, the application of competing-risks analysis enhances the relevance and reliability of the results, as it comprehensively accounts for the complexities inherent in real-world scenarios. Hence, we posit that the outcomes derived from the competing-risks analysis are likely to offer superior accuracy.

Socioeconomic factors can affect the health level of individuals and the allocation of medical resources, thereby affecting the treatment methods and effects received by patients. At the same time, due to the shortage of medical resources, socio-economic factors also affect patients' diagnosis and treatment time and treatment choices to a certain extent. The relationship between income and prognosis of cancer patients is a complex issue, but the current research results are not consistent, and further research is necessary. Many studies have considered income to be important influencing factor for the prognosis of patients with cSCC, because income can affect the treatment plan (including methods and drugs applied) and other factors of patients. Most studies have found higher income to be related to better patient prognoses [37–41]. We hypothesize that the influence of income on survival time remains relatively constant and does not exhibit significant time-dependent variations. Consequently, the Cox regression model may offer a more precise representation of this influence. This observation could elucidate why the Cox model yields a smaller HR value and a narrower 95% confidence interval when analyzing the income variable. Competing-risks analysis indicated that income was a factor affecting prognosis of patients, but the prognosis of the high-income group and mediate-income group were actually worse than that of the low-income group (mediate income: HR = 17.596, CI = 2.431–127.369, *P* ≤ 0.05; high income: HR = 13.939, CI = 1.858–104.587, *P* ≤ 0.05). We believed that this interesting phenomenon may be related to the lifestyle, cognition, environment, and other factors of different income groups [42]. High income does not necessarily mean having a good lifestyle and cognitive attitude, but a good lifestyle and attitude can have an important beneficial impact on the prognosis of the disease. Studies have found that income has little effect on health risk, but higher income is associated with a higher risk of abnormal BMI in males [43]. Relevant literature [44–47] also suggests that high-income people tend to face greater work and life pressures. Unhealthy lifestyle and mental and psychological factors will also follow, which in turn affect the disease prognosis of these patients. This result may also be related to the patients' baseline health status, age distribution, etc., and other variables that may affect the prognosis were not included, resulting in confounding. These differences may affect the comparison of outcomes [48]. We believe that it is necessary to perform a multilevel and in-depth analysis in the future to explore the impacts of the above factors on the prognosis of patients with micSCC.

Factors such as gender, race, AJCC stage, surgery, radiotherapy, and chemotherapy did not reach statistical significance in either the Cox regression model or the Fine-Gray model. However, it's imperative to consider that the context of rare malignancies like micSCC presents unique challenges in drawing definitive prognostic conclusions. Discrepancies between these findings and prior studies on cSCC could stem from differences in study design, sample selection, or data quality. This underscores the challenge presented by the scarce research dedicated to micSCC, emphasizing the intricacy of prognosis determination, especially considering our dependence on established prognostic factors shared with cSCC. Moreover, the ongoing debates within the medical community concerning therapeutic modalities in managing squamous cell carcinoma underscore the intricate interplay among various clinical and patient-specific variables [49, 50]. In this study, our approach mirrored the common practice among SEER database studies for managing missing data. However, this methodology might have influenced results by reducing sample size, limiting statistical power, and potentially missing relevant associations. Analyzing demographic profiles revealed significant differences, notably higher average age and increased male and white representation among excluded patients. This imbalance suggests a potential for selection bias, exacerbated by a significant amount of missing data, complicating accurate comparisons and underscoring the need for careful consideration of their impact. Despite these challenges, we maximized available data for analysis to minimize biases' potential effects on our conclusions.

This was the first study of the prognostic factors of patients with micSCC. There have been few previous studies on the prognostic factors of cSCC and its subtypes, and most of them used Cox and logistic regression analyses. Most of the studies of the prognosis of patients with cSCC patients reported on the Internet were based on single or several factors, with the significant factors including age, poor differentiation, perineural invasion, tumor size and depth, and immunosuppression [27, 28, 31, 35, 36, 51]. Accurate prognostic factors research will be more conducive to clinicians to carry out individualized treatment for micSCC patients, provide more favorable treatment options for patient survival, evaluate curative effect, and guide clinical follow-up treatment [27]. In our study, we assumed that these two competing risks are independent, which means that the occurrence of one event will not affect the probability of the other event, such as patient suicide or death from cardiovascular disease. The plausibility of this assumption is based on our understanding of the disease process. Death caused by micSCC and death caused by other causes are two distinct outcomes, and their occurrence is driven by different biological mechanisms. We believe that the assumption of independence between these two events is reasonable. However, we also realize that this is a strong assumption and may not always hold. For example, if a patient's micSCC worsens, they may be more likely to die from other health problems. Therefore, we have considered factors that may affect the independence of competing risks as much as possible in our analysis, including patients' age, gender, marital status, etc. We look forward to further exploring this issue in future work to improve the accuracy and stability of our model. Future research may also include sensitivity analyzes or alternative models that relax assumptions to account for the complexity of potential dependencies among competing risks. As research on micSCC remains relatively scarce, future investigations will warrant the aggregation of more extensive datasets to facilitate deeper and more rigorous analyses.

## Limitations

The limitations of this study must not be overlooked. First, the duration of data collection selected in this study was relatively short (2000–2015). Because the overall prognosis of micSCC is good after early treatment, the occurrence of death from a specific cause is low over a short period of time, which may lead to a small number of samples. Second, selection bias was inevitable given the retrospective nature of this study. Third, we did not evaluate some aspects such as vascular metastasis, family history, biomolecular markers, or other histological results. Fourth, the absence of an exploration into potential interactions between risk factors is another limitation that may influence outcomes. Fifth, using the traditional data processing method of listwise deletion to deal with missing data may introduce sample selection bias. Finally, as with any statistical model, there is a potential risk of model misspecification.

## Conclusions

In summary, this study is the first to uncover that, in the context of competing risks, age, tumor size, and income serve as independent risk factors influencing the risk of mortality due to micSCC among patients. These findings hold implications for clinicians, aiding them in refining patient prognoses and making informed clinical decisions for personalized treatment strategies. Furthermore, our results contribute to the foundation for future research endeavors focused on advancing our understanding of micSCC and its complexities. As a next step, prospective studies could explore the interplay of these risk factors in larger and more diverse patient cohorts, further elucidating their combined influence on micSCC outcomes. Furthermore, investigating potential biomarkers associated with these risk factors might unveil novel avenues for early detection and more precise interventions.

### Supplementary Information

**Additional file 1.**

## Data Availability

The datasets utilized and analyzed in the study were acquired from the SEER database and were in compliance with the relevant requirements. The data for this article comes from https://seer.cancer.gov/.
