# Supplementary material for: Competing-risks analysis for evaluating the prognosis of patients with microinvasive cutaneous squamous cell carcinoma based on the SEER database
Source: BMC Med Res Methodol. 2023 Dec 7;23:286. doi: 10.1186/s12874-023-02109-x (PMC10701925; doi:10.1186/s12874-023-02109-x)
Supplement: Supplementary file 1 — Additional file 1. [file 12874_2023_2109_MOESM1_ESM.docx]

Supplementary Material

correlation coefficient matrices：

Age Sex Race Marital AJCC_stage stage

Age 1.0000000000 -0.280884586 0.040206191 0.156588978 0.143615811 0.10081874

Sex -0.2808845859 1.000000000 0.036385828 -0.009991657 -0.184827654 -0.11668512

Race 0.0402061908 0.036385828 1.000000000 -0.008359088 0.009743139 0.01372280

Marital 0.1565889777 -0.009991657 -0.008359088 1.000000000 0.052195120 0.01984042

AJCC_stage 0.1436158114 -0.184827654 0.009743139 0.052195120 1.000000000 0.83740198

stage 0.1008187404 -0.116685119 0.013722798 0.019840425 0.837401981 1.00000000

Radiation -0.1619523586 0.219376263 0.004001562 -0.083364149 -0.537584217 -0.52994289

Chemotherapy -0.0756471405 0.196545565 -0.013910759 -0.068980872 -0.604708479 -0.60896144

Surg 0.0983415236 -0.208206672 -0.004264421 0.103825465 0.487366669 0.48550909

Reg_LN_surg -0.0008793497 -0.020072537 -0.027550590 0.016402389 -0.038293018 -0.05271685

Size 0.0981094031 -0.091537673 0.040887955 0.053178956 0.627676092 0.53403915

RNE 0.0217039282 -0.049483708 -0.037017017 0.029672692 -0.065387184 -0.06776971

income 0.0359886815 -0.012984200 0.087575689 -0.050762981 -0.049811926 -0.04548894

COD 0.3604919488 -0.139808004 -0.015162983 0.151903238 0.174598444 0.12610819

Survival_months -0.1914955639 0.172107567 0.028600488 -0.076305592 -0.141797969 -0.12344660

OS 0.3626454282 -0.155149457 -0.017372699 0.161163274 0.248426219 0.19550118

MFDTT 0.0918740511 -0.030626166 -0.010217780 0.013201733 0.099109611 0.08806111

Radiation Chemotherapy Surg Reg_LN_surg Size RNE

Age -0.161952359 -0.075647141 0.098341524 -0.0008793497 0.09810940 0.021703928

Sex 0.219376263 0.196545565 -0.208206672 -0.0200725371 -0.09153767 -0.049483708

Race 0.004001562 -0.013910759 -0.004264421 -0.0275505903 0.04088795 -0.037017017

Marital -0.083364149 -0.068980872 0.103825465 0.0164023890 0.05317896 0.029672692

AJCC_stage -0.537584217 -0.604708479 0.487366669 -0.0382930182 0.62767609 -0.065387184

stage -0.529942889 -0.608961443 0.485509087 -0.0527168540 0.53403915 -0.067769715

Radiation 1.000000000 0.771288570 -0.662045244 -0.0050989265 -0.52845844 -0.019689280

Chemotherapy 0.771288570 1.000000000 -0.670851127 0.0034418075 -0.64665341 -0.012486139

Surg -0.662045244 -0.670851127 1.000000000 -0.0035019116 0.51932122 0.026981211

Reg_LN_surg -0.005098926 0.003441808 -0.003501912 1.0000000000 -0.01078053 0.768164170

Size -0.528458443 -0.646653411 0.519321217 -0.0107805289 1.00000000 -0.020512573

RNE -0.019689280 -0.012486139 0.026981211 0.7681641702 -0.02051257 1.000000000

income 0.068453350 0.048451906 -0.012034780 0.0349322843 -0.02577050 -0.004199653

COD -0.218343051 -0.136878656 0.155613181 0.0444672264 0.11109999 0.051074850

Survival_months 0.183405465 0.146281119 -0.167058785 0.0605613987 -0.11856242 -0.078213954

OS -0.272979894 -0.210115652 0.204991320 0.0320018541 0.17923434 0.042543804

MFDTT -0.123722400 -0.098756096 0.156077976 -0.0239017745 0.11429868 -0.034224548

income COD Survival_months OS MFDTT

Age 0.035988681 0.360491949 -0.19149556 0.36264543 0.091874051

Sex -0.012984200 -0.139808004 0.17210757 -0.15514946 -0.030626166

Race 0.087575689 -0.015162983 0.02860049 -0.01737270 -0.010217780

Marital -0.050762981 0.151903238 -0.07630559 0.16116327 0.013201733

AJCC_stage -0.049811926 0.174598444 -0.14179797 0.24842622 0.099109611

stage -0.045488939 0.126108191 -0.12344660 0.19550118 0.088061113

Radiation 0.068453350 -0.218343051 0.18340547 -0.27297989 -0.123722400

Chemotherapy 0.048451906 -0.136878656 0.14628112 -0.21011565 -0.098756096

Surg -0.012034780 0.155613181 -0.16705878 0.20499132 0.156077976

Reg_LN_surg 0.034932284 0.044467226 0.06056140 0.03200185 -0.023901775

Size -0.025770503 0.111099990 -0.11856242 0.17923434 0.114298675

RNE -0.004199653 0.051074850 -0.07821395 0.04254380 -0.034224548

income 1.000000000 -0.029470108 0.14167785 -0.01536636 -0.026157589

COD -0.029470108 1.000000000 -0.28471579 0.96433128 0.008430421

Survival_months 0.141677853 -0.284715792 1.00000000 -0.30928188 -0.021821949

OS -0.015366362 0.964331276 -0.30928188 1.00000000 0.014751156

MFDTT -0.026157589 0.008430421 -0.02182195 0.01475116 1.000000000

variance inflation factors (VIF)：

Variable VIF

1 Age 1.160568

2 Sex 1.160568

3 Race 1.160568

4 Marital 1.160568

5 AJCC_stage 1.160568

6 stage 1.160568

7 Radiation 1.160568

8 Chemotherapy 1.160568

9 Surg 1.160568

10 Reg_LN_surg 1.160568

11 Size 1.160568

12 RNE 1.160568

13 income 1.160568

14 MFDTT 1.160568

Parameter estimation of Fine-Gray model：

Competing Risks Regression

Call:

crr(ftime = mydata_fg$ftime, fstatus = mydata_fg$fstatus, cov1 = mydata_fg[,

c("Age", "Sex", "Race", "Marital", "AJCC_stage", "stage",

"Radiation", "Chemotherapy", "Surg", "Reg_LN_surg", "Size",

"RNE", "income", "MFDTT")])

coef exp(coef) se(coef) z p-value

Age 0.0447 1.046 0.0165 2.708 0.0068

Sex -0.5582 0.572 0.7450 -0.749 0.4500

Race -0.4188 0.658 0.3745 -1.118 0.2600

Marital 0.4383 1.550 0.2523 1.737 0.0820

AJCC_stage 0.2992 1.349 0.5736 0.522 0.6000

stage 0.4868 1.627 1.0465 0.465 0.6400

Radiation -0.9200 0.399 1.2686 -0.725 0.4700

Chemotherapy -0.1871 0.829 1.1912 -0.157 0.8800

Surg -0.1850 0.831 0.9132 -0.203 0.8400

Reg_LN_surg -0.7621 0.467 0.9965 -0.765 0.4400

Size 0.8012 2.228 0.3689 2.172 0.0300

RNE 0.4413 1.555 0.9210 0.479 0.6300

income 0.4928 1.637 0.2019 2.441 0.0150

MFDTT -0.1634 0.849 0.1972 -0.829 0.4100

exp(coef) exp(-coef) 2.5% 97.5%

Age 1.046 0.956 1.0124 1.08

Sex 0.572 1.748 0.1329 2.46

Race 0.658 1.520 0.3157 1.37

Marital 1.550 0.645 0.9452 2.54

AJCC_stage 1.349 0.741 0.4382 4.15

stage 1.627 0.615 0.2092 12.65

Radiation 0.399 2.509 0.0332 4.79

Chemotherapy 0.829 1.206 0.0803 8.56

Surg 0.831 1.203 0.1388 4.98

Reg_LN_surg 0.467 2.143 0.0662 3.29

Size 2.228 0.449 1.0813 4.59

RNE 1.555 0.643 0.2557 9.45

income 1.637 0.611 1.1021 2.43

MFDTT 0.849 1.178 0.5770 1.25

Num. cases = 1259

Pseudo Log-likelihood = -212

Pseudo likelihood ratio test = 101 on 14 df,

Model diagnosis using crskdiag package：

***P-values for model diagnostics***

Linear functional form for a single covariate:

Age Sex Race Marital AJCC_stage stage Radiation

0.583 1.000 0.801 0.137 0.208 0.256 1.000

Chemotherapy Surg Reg_LN_surg Size RNE income MFDTT

1.000 1.000 1.000 0.971 1.000 0.018 0.913

Link function:

[1] 0.281

[1] "Results for 'prop' test:"

***P-values for model diagnostics***

Proportionality for a single covariate:

Age Sex Race Marital AJCC_stage stage Radiation

0.424 0.383 0.719 0.128 0.486 0.871 0.359

Chemotherapy Surg Reg_LN_surg Size RNE income MFDTT

0.206 0.776 0.612 0.022 0.240 0.855 0.181

Overall proportionality:

[1] 0.473
